# Chromatin remodeler CHD7 targets active enhancer region to regulate cell type-specific gene expression in human neural crest cells

**DOI:** 10.1038/s41598-022-27293-6

**Published:** 2022-12-31

**Authors:** Tsukasa Sanosaka, Hironobu Okuno, Noriko Mizota, Tomoko Andoh-Noda, Miki Sato, Ryo Tomooka, Satoe Banno, Jun Kohyama, Hideyuki Okano

**Affiliations:** Department of Physiology, Keio University School of Medicine, 35 Shinanomachi, Shinjuku-ku, Tokyo, 160-8582 Japan

**Keywords:** Chromatin remodelling, Mesenchymal stem cells, Gene regulation

## Abstract

A mutation in the chromatin remodeler chromodomain helicase DNA-binding 7 (CHD7) gene causes the multiple congenital anomaly CHARGE syndrome. The craniofacial anomalies observed in CHARGE syndrome are caused by dysfunctions of neural crest cells (NCCs), which originate from the neural tube. However, the mechanism by which CHD7 regulates the function of human NCCs (hNCCs) remains unclear. We aimed to characterize the cis-regulatory elements governed by CHD7 in hNCCs by analyzing genome-wide ChIP-Seq data and identifying hNCC-specific CHD7-binding profiles. We compared CHD7-binding regions among cell types, including human induced pluripotent stem cells and human neuroepithelial cells, to determine the comprehensive properties of CHD7-binding in hNCCs. Importantly, analysis of the hNCC-specific CHD7-bound region revealed transcription factor AP-2α as a potential co-factor facilitating the cell type-specific transcriptional program in hNCCs. CHD7 was strongly associated with active enhancer regions, permitting the expression of hNCC-specific genes to sustain the function of hNCCs. Our findings reveal the regulatory mechanisms of CHD7 in hNCCs, thus providing additional information regarding the transcriptional programs in hNCCs.

## Introduction

Cell type-specific transcriptional responses regulate cellular identity, which is dynamically coordinated during the developmental process^1^. Enhancers are genetic elements that were historically defined as DNA sequences that potentiate the cell type-specific transcription of transcriptional start sites (TSSs) located distally to the enhancer regions^2^. Although enhancers are responsible for exerting cell type-specific gene expression, they need to be accessible by adapting the local chromatin structure and enabling a precise response to external stimuli during development^3,4^. Accordingly, chromatin-based epigenetic alterations are closely linked to enhancer activity. The importance of chromatin regulation is also evidenced by the fact that defects in chromatin regulators cause genetic developmental disorders called chromatinopathies^5,6^. Notably, a genetic mutation in chromatin remodelers was observed in a subset of chromatinopathies, including ATRX syndrome, Floating-Harbor syndrome, and CHARGE syndrome^5^.

CHARGE syndrome is a congenital disorder caused by a heterozygous mutation in *CHD7*^7,8^. CHD7 is a member of the chromodomain helicase DNA-binding (CHD) family, a group of ATP-dependent chromatin-remodeling factors that alter chromatin structure by rearranging the position and organization of nucleosomes on DNA^9^.

We previously demonstrated an epigenetic landscape regulated by CHD7 in human induced pluripotent stem cell (hiPSC)-derived neuroepithelial cells (hNECs)^10^. Notably, CHD7 binds and regulates the activity of central nervous system-specific enhancers to control the differentiation capacity of hNECs^10^, indicating an essential role of CHD7 in regulating enhancers, at least in the central nervous system.

Dysfunction of neural crest cells (NCCs) is a primary cause of CHARGE syndrome^8^, and the role of CHD7 in regulating the epigenetic signature of NCCs has thus been of interest. The pioneering work by Wysocka’s team using an in vitro differentiation system from human embryonic stem cells demonstrated that the formation of NCCs required the function of CHD7^11^. Additionally, we previously demonstrated defective migration of human NCCs (hNCCs) derived from iPSCs from patients with CHARGE syndrome^12^. These results provide potential explanations for the pathology of CHARGE syndrome. However, although CHD7 has been shown to be essential for the function and development of hNCCs, the comprehensive genomic map governed by CHD7 in hNCCs is still unclear.

We previously optimized the experimental conditions of chromatin immunoprecipitation (ChIP) followed by massive parallel DNA sequencing (ChIP-seq) for CHD7 and successfully generated CHD7-binding profiles in hNECs^10^. However, detailed information on the cell type-specific role of CHD7, especially in hNCCs, was not determined. In the current study, we aimed to compare CHD7-binding profiles in hiPSCs, hNECs, and hNCCs to identify hNCC-specific targets of CHD7. These findings highlight the fundamental role of CHD7 in cell type-specific gene regulation in hNCCs.

## Results

### Identification of cell type-specific CHD7 targets by ChIP-seq in hiPSCs, hNECs, and hNCCs

**Figure 1:**
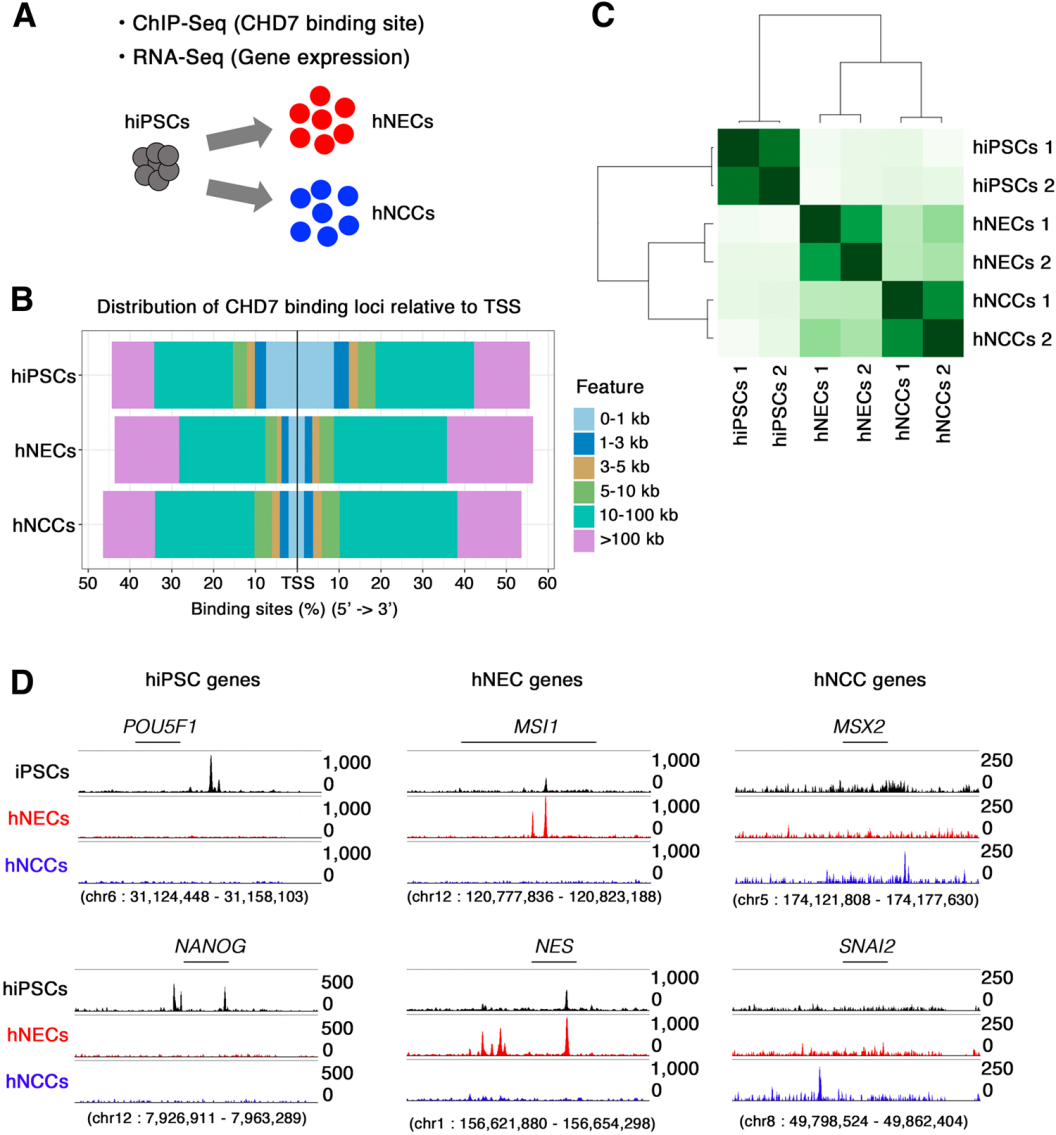
Identification of cell type-specific CHD7 targets. (**A**) Schematic illustration of the current study. (**B**) Distribution of CHD7-binding loci from TSS regions in hiPSCs, hNECs, and hNCCs. (**C**) Hierarchical clustering using Pearson’s correlation analysis across the entire sample set. (**D**) ChIP-seq signals for CHD7 at genomic loci specific to hiPSCs (*POU5F1* and *NANOG*) (left panel), hNECs (*MSI1* and *NES*) (middle panel), and hNCCs (*MSX2* and *SNAI2*) (right panel) in each cell line.

The pivotal function of CHD7 in the development and function of various tissues depends on the cellular context^10,11,13–18^, and identifying the cell-specific targets of CHD7 is thus necessary to elucidate its function. We therefore investigated the function of CHD7 in hNCCs using the ChIP-seq profiles of CHD7 in hiPSCs, hNECs, and hNCCs^10^ with transcriptome information using published datasets (Fig. 1A and Supplementary Table S1). We initially identified the CHD7-binding profiles using the Model-based Analysis of the ChIP-Seq (MACS2) algorithm and examined the distribution of CHD7-binding peaks in the genome (Fig. 1B and Supplementary Fig. S1). The average distribution of CHD7 peaks displayed similar genomic distributions, peaking at the TSS and transcription end site (TES) in all the tested cells in the current analysis (Supplementary Fig. S1). Most CHD7-binding peaks were located distally from the TSS in hNECs and hNCCs (Fig. 1B), but were preferentially located in the proximal region of the TSS in hiPSCs. These results indicated that the involvement of CHD7 in gene regulation is cell-type dependent (Fig. 1B). We further selected cell type-specific CHD7-binding peaks using DiffBind^19^. Hierarchical clustering analysis of CHD7-binding regions revealed differential CHD7 occupancy among hiPSCs, hNECs, and hNCCs (Fig. 1C). We also found clear separation of cell type-specific CHD7 peaks in representative gene loci (Fig. 1D).

### CHD7 preferentially bound to active enhancers in hNCCs

**Figure 2:**
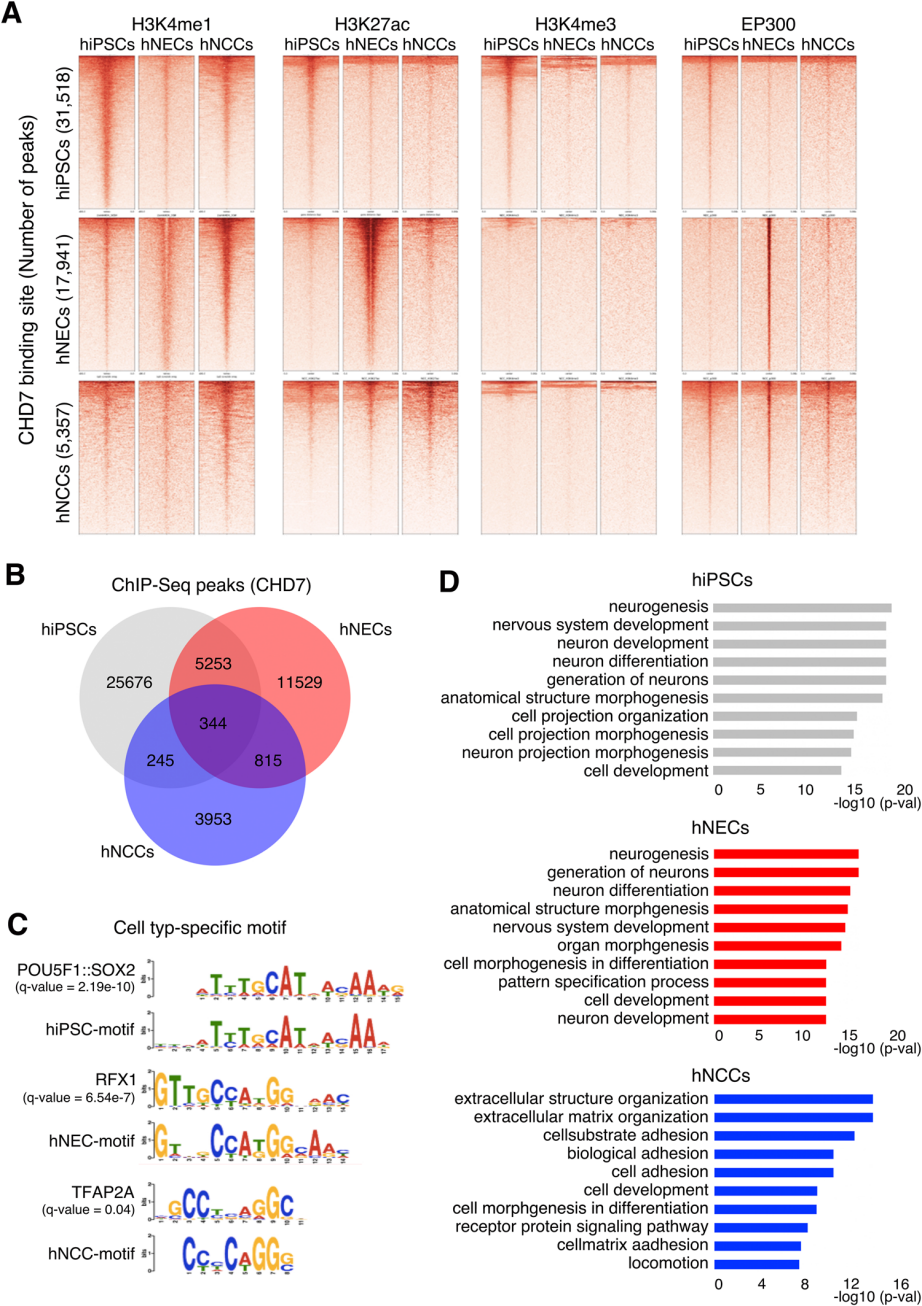
Cell type-specific functions of CHD7. (**A**) Heatmap analyses of ChIP-seq signal of EP300 and selected histone modifications in hiPSCs, hNECs, and hNCCs (H3K4me1, H3K4me3, and H3K27ac). Read counts were extracted for all ChIP-seq experiments within a region spanning ± 5 kb around the center of each annotated CHD7-peaks detected in each cell type (1st row: 31,518 peaks in hiPSCs, 2nd row: 17,941 peaks in hNECs and 3rd row: 5357 peaks in hNCCs). (**B**) Overlaps of CHD7-binding regions across different cell types. (**C**) Cell type-specific CHD7-binding sequences were analyzed using the top 500 binding regions of CHD7-ChIP-seq data. The detected sequences were compared using the JASPAR motif database. (**D**) GO analysis for biological processes associated with CHD7-bound regions in hiPSCs, hNECs, and hNCCs. Bar plots displaying the top 10 GO categories.

To gain further insights into the functional properties of CHD7, we cross-referenced CHD7-bound regions with the genome-wide chromatin state of histone modification in hiPSCs, hNECs, and hNCCs (Fig. 2A). We generated chromatin landscapes of cell type-specific CHD7-bound regions, including H3K4me1, H3K27ac, H3K4me3, and EP300. Bioinformatics analyses revealed a striking overlap between CHD7-bound regions and p300, H3K27ac, and H3K4me1, as markers indicative of active enhancers in hNECs and hNCCs (Fig. 2A). In contrast, CHD7 binding in hiPSCs was most likely to occur within the promoter region, evidenced by the correlation with H3K4me3, as described previously^20,21^. We aimed to identify the cell type-specific function of CHD7 and we therefore compared the locations of ChIP-seq peaks among hiPSCs, hNECs, and hNCCs. Although there were some shared peaks, most peaks were specific for each cell type (25,676 (81.5%) of 31,518 peaks in hiPSCs, 11,529 (64.3%) of 17,941 peaks in hNECs, and 3,953 (73.8%) of 5,357 peaks in hNCCs) (Fig. 2B and Supplementary Fig. S1). A total of 1,159 peaks were shared between hNECs and hNCCs, and 16,782 and 4,198 peaks were specific to hNECs and hNCCs, respectively, suggesting that CHD7 might play cell type-specific roles (Fig. 2B). CHD7 functions as a chromatin remodeler and requires interaction with DNA-bound transcription factors^9^. We thus speculated that the detected binding sites might aid the detection of such transcription factors in each cell type. We therefore performed motif analysis using MEME software to define cell type-specific peaks in each cell type^22,23^ (Supplementary Fig. S1). The known binding motifs that matched the de novo motif in the cell type-specific peaks were target sequences for POU5F1:SOX2^24^ in hiPSCs, regulatory factor X1^25^ in hNECs, and transcription factor AP-2α (TFAP2A)^26^ in hNCCs, respectively (Fig. 2C); notably, each transcription factor is a known regulator of each cell type. For hNCCs, TFAP2A is required for both neural plate-border induction and specification of NCCs^26^. TFAP2A also acts as a pioneer transcription factor that engages silent genes, helping to initiate neural crest (NC) specification^27^. As noted above, dysfunction of hNCCs has been reported to be involved in the pathogenesis of CHARGE syndrome^12,28,29^. A potential link between CHD7 and TFAP2A might thus provide potential mechanistic insights into the pathogenesis of CHARGE-derived hNCCs.

Gene Ontology (GO) analysis for hiPSCs and the hNEC-specific CHD7-regulated region revealed a similar trend, indicating that the sites were associated with neural differentiation, including “neurogenesis,” “nervous system development,” and “neuron differentiation” (Fig. 2D). In contrast, hNCC-specific CHD7-regulated regions were associated with mesenchymal properties of the NC, including “extracellular structure organization,” “extracellular matrix organization,” and “biological adhesion,” indicating successful identification of cell type-specific CHD7-bound regions in the current analyses (Fig. 2D).

### CHD7 binding predicted cell type-specific gene expression in hNCCs

**Figure 3:**
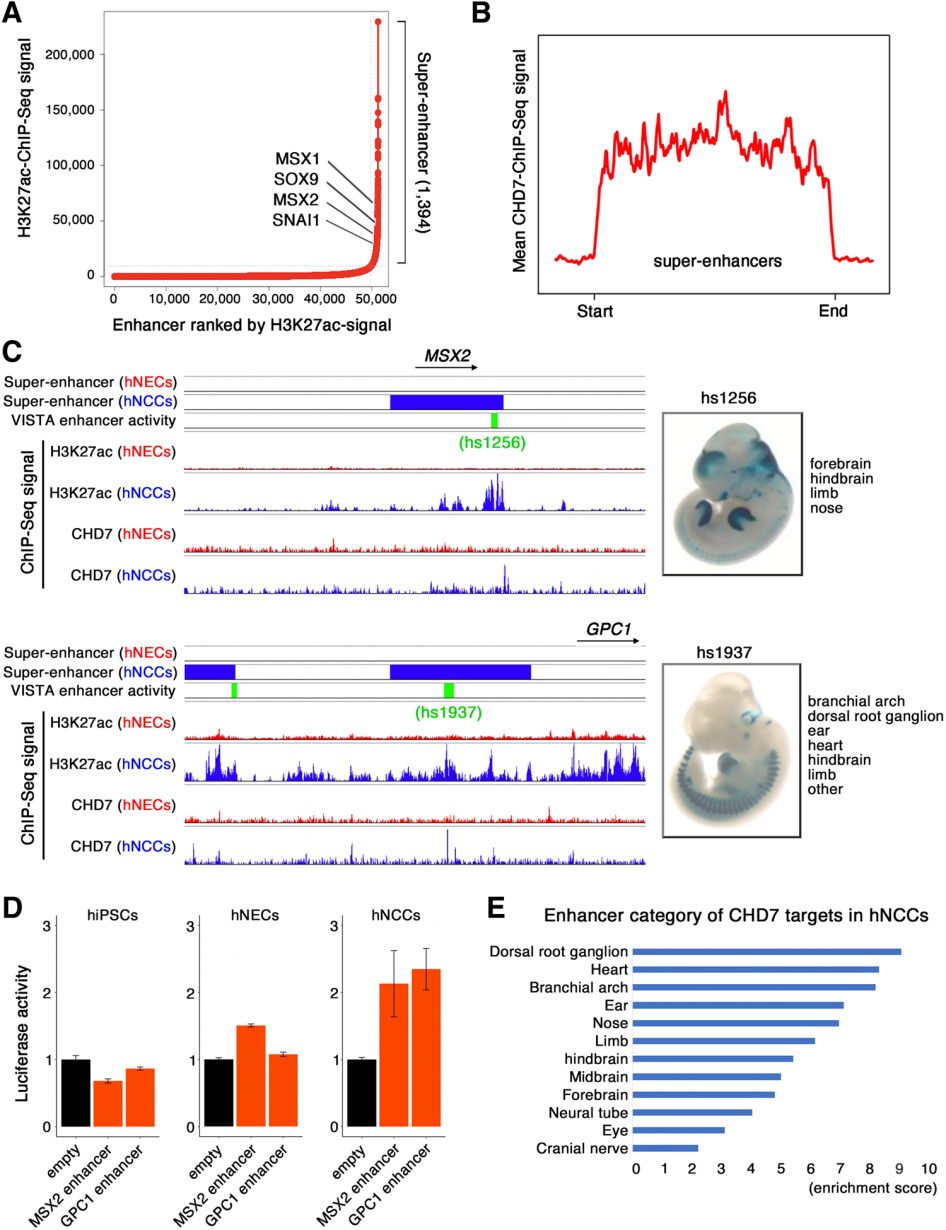
CHD7 bound to SE regions with TFAP2A and NR2F1/2. (**A**) Distribution of H3K27ac ChIP-seq density at enhancers in increasing order of input-normalized H3K27ac ChIP-seq signal. Enhancers above the curve’s inflection point are considered SEs. The respective ranks of SEs and their associated genes are shown. (**B**) Density plots of mean CHD7 ChIP-seq signals across SEs in hNCCs. (**C**) Snapshots showing combined tracks of SEs (red; hNEC, blue; hNCC), VISTA enhancer activity (green), and ChIP-seq data (α-H3K27ac and α-CHD7) near *MSX2* and *GPC1*. Images on right are screenshots of tissue-specific activities of the enhancer proximal to *MSX2* and *GPC1* tested in a lacZ reporter transgenic mouse assay extracted from the VISTA Enhancer Browser database (https://enhancer.lbl.gov/). (**D**) A luciferase reporter assay was performed to determine the activity of CHD7-bound enhancers near *MSX2* and *GPC1*. Reporter plasmid (empty control, *MSX2* enhancer, and *GPC1* enhancer) was transfected to hiPSCs, hNECs, and hNCCs to compare enhancer activity. Renilla reporter was co-transfected for normalization (F/R ratio). Y axis shows fold enrichment over an empty control. Data are presented as the mean ± SEM. (**E**) Functional analysis of genomic regions of CHD7-bound regions intersected with enhancer regions validated in the VISTA Enhancer Browser database in hNCCs. Bar plot showing enrichment of enhancer occupancy across 16 different tissues.

A previous study showed that super-enhancers (SEs) conferred cellular identities by regulating tissue-specific gene expression^1,30,31^. Moreover, we previously identified CHD7 as one of the components enriched in SEs across the hNEC genome^10^. This prompted us to determine if CHD7 was also enriched in SEs in hNCCs. We delineated SEs in hNCCs based on H3K27ac abundance using the ROSE algorithm, and identified 1,394 SEs (Fig. 3A). The identified SEs were located proximal to genes encoding key regulators of hNCC development, including *SOX9*, *MSX1*, and *MSX2*^32–34^ (Fig. 3A, Supplementary Table S2). Furthermore, we found that CHD7 bound to SEs in hNCCs (Fig. 3B). To further evaluate the enhancer activity of CHD7-bound regions, we cross-referenced these regions with the VISTA Enhancer Browser^35^, a database of tissue-specific human enhancers with validated activity in mice. For example, CHD7 binding at SE-associated genes in hNCCs, including *MSX2*^36^ and *GPC1*^37^loci, coincided with the presence of annotated enhancers that exhibited intense activity in NCC-associated tissues (Fig. 3C). To examine enhancer activity of these two loci in hNCCs, we generated luciferase reporter system containing the predicted enhancer region of *MSX2* or *GPC1* (Fig. 3D). hiPSCs, hNECs, and hNCCs were transfected with the luciferase reporter. As a reference, these cells were transfected with an empty vector. Then, the enhancer activity was evaluated by the degree of increased reporter activity with respect to the empty vector. As expected, higher reporter activity was detected in hNCCs compared to hiPSC and hNECs (Fig. 3D). In addition, the VISTA Enhancer Browser showed that CHD7-binding sites were most enriched for regions that displayed enhancer activity in NC derivatives, including dorsal root ganglia, heart, and ear (Fig. 3E). The identification of hNCC-specific CHD7-enriched enhancers suggests that CHD7 regulates hNCC-specificity of its corresponding genes.

**Figure 4:**
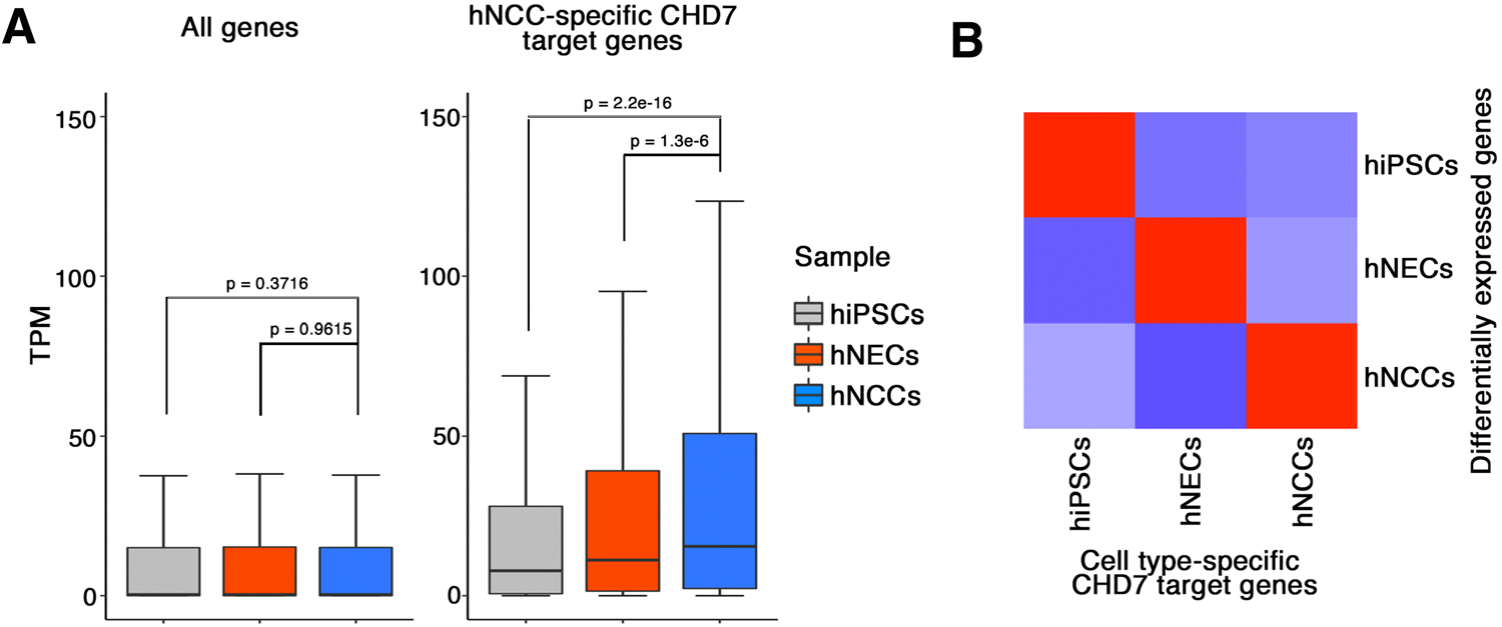
Correlation of CHD7-bound enhancer regions with transcription of target genes in hNCCs. (**A**) Boxplot showing expression of all genes (left) and hNCC-specific CHD7-target genes (right) in hiPSCs, hNECs, and hNCCs. (**B**) Correlation analysis of cell type-specific CHD7 target genes and DEGs in hiPSCs, hNECs, and hNCCs. Heatmap showing z-score of correlations.

We further elucidated the role of CHD7 in transcriptional regulation by examining the expression of cell type-specific CHD7 target genes. First, we determined cell type-specific CHD7 target genes based on the ChIP-seq information for hiPSCs, hNECs, and hNCCs (Supplementary Table S3). We then examined the expression of hNCC-specific CHD7 target genes in each cell type. As expected, the expression levels of these genes were higher in hNCCs (Fig. 4A). We also performed a correlation analysis and showed that the expression of cell type-specific CHD7 target genes was positively correlated with the expression of cell type-specific genes (Fig. 4B).

### Co-occupancy of CHD7 with TFAP2A and nuclear receptor subfamily 2 group F1/F2 (NR2F1/F2)

**Figure 5:**
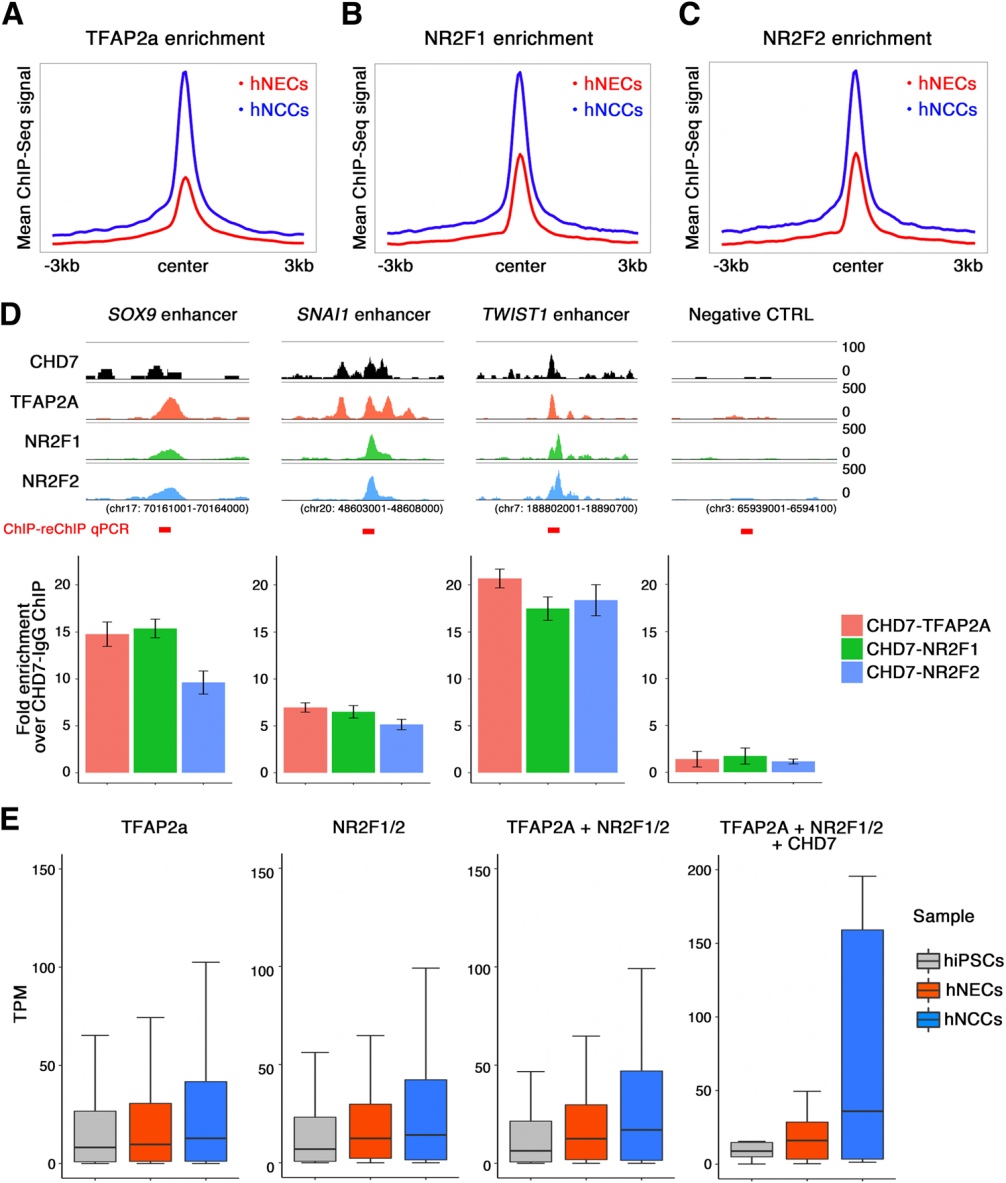
Co-localization of CHD7, TFAP2A, and NR2F1/2 strongly induced hNCC- specific gene expression. (**A**) Enrichment analysis of TFAP2A in hNECs and hNCCs. The mean TFAP2A ChIP-seq signal from ± 3 kb of the CHD7-bound center was plotted. (**B**) Enrichment analysis of NR2F1 around CHD7-bound regions in hNECs and hNCCs. The mean NR2F1-ChIP-seq signal was plotted. (**C**) Enrichment analysis of NR2F2 around CHD7-bound regions in hNECs and hNCCs. The mean NR2F2-ChIP-seq signal was plotted. (**D**) ChIP-reChIP analysis of CHD7-TFAP2A-NR2F1/2 co-occupancy. Snapshot of ChIP-Seq signal (CHD7: black, TFAP2A: red, NR2F1: green, NR2F2: blue) in hNCC enhancer region of NCC specific genes. The genomic region indicated the whole loci shown in the snapshot. The red bar indicates the amplified region at ChIP-reChIP qPCR analysis. ChIP-reChIP analysis was performed using an anti-CHD7 antibody as the first antibody. Antibodies against control IgG, TFAP2A (red), NR2F1 (blue), or NR2F2 (green) antibodies were used for reChIP, respectively. Immunoprecipitated DNA was analyzed by qPCR of selected hNCC enhancers shown in the upper panels. Y axis shows fold enrichment over a CHD7-IgG ChIP-reChIP. (**E**) Target genes of CHD7, TFAP2A, and NR2F1/2 were extracted and gene expressions in each cell type were plotted.

The master NC regulators TFAP2A and NR2F1/F2 have been reported to be involved in the hNCC enhancer regulatory mechanism^38^. Given that motif analysis of the CHD7-binding region in hNCCs revealed the possible co-existence of TFAP2A with CHD7 (Fig. 2C), we analyzed the relationship among CHD7, TFAP2A, and NR2F1/F2 in hNCCs. Reanalysis of published datasets for TFAP2A, NR2F1, and NR2F2 ChIP-seq experiments in hNCCs^38^ showed that TFAP2A bound preferentially to the proximal region of its targets. Additionally, its bound regions were associated with H3K4me3 and EP300, indicative of the role of TFAP2A as a direct transcriptional activator (Supplementary Fig. S2). In contrast, the NF2F1/F2-binding datasets showed that a substantial fraction of NR2F1/2 bound to the distal regions of their targets with increased enhancer activity, demarcated by co-occupancy of H3K4me1 and H3K27ac (Supplementary Fig. S2). We investigated the interplay of CHD7 with TFAP2A and NR2F1/2 in hNCCs by examining the enrichment of TFAP2A and NR2F1/2 in the CHD7-binding region in hNCCs. As a reference, we also compared their enrichment in the CHD7-binding region in hNECs. Interestingly, these transcription factors were preferentially enriched in hNCC-specific CHD7-binding regions, and their abundance was substantially lower in hNEC-specific CHD7-binding regions (Fig. 5A–C). These results indicate that the co-existence of tissue-specific transcription factors ensures cell type-specific CHD7 binding. A previous study^38^ showed that hNCC-specific gene expression was linked to TFAP2A- or NR2F1/2-binding regions. In particular, it has been shown that TFAP2A and NR2F1/2 are present in the enhancer regions of hNCC genes such as *SOX9*, *SNAI1*, and *TWIST1*, which are essential genes for neural crest induction and differentiation^39^. Notably, these genes are also targeted by CHD7 in hNCCs (Fig. 5D). To directly examine whether CHD7 actually interacts with TFAP2A and NR2F1/2 in hNCC-specific CHD7 targets, we performed sequential ChIP experiments called ChIP-reChIP experiments^40^ (Fig. 5D). ChIP-ReChIP-qPCR analysis showed enrichment of CHD7-TFAP2A, CHD7-NR2F1, and CHD7-NR2F2 signals over the CHD7-IgG signal at enhancers, but not at control regions (Fig. 5D). These observations indicated co-occupancy of CHD7, TFAP2A, and NR2F1/2 on CHD7-targeted regions in hNCC. Furthermore, to investigate the impact of the co-occupancy of these factors on their targets in hNCCs, we examined the gene expression classified by the degree of binding of each factor (Fig. 5E). Interestingly, genes co-occupied by CHD7 together with TFAP2A and NR2F1/2 further elaborated hNCC-specific gene expression, indicating the synergistic actions of these factors as a basis for cell type-specific transcription in hNCCs (Fig. 5E and Supplementary Fig. S4A).

## Discussion

This study provides the first genome-wide mapping of CHD7 in hNCCs. Combined with chromatin-state profiling, it revealed that CHD7 acts as an activator of enhancers to specify hNCC-specific gene expression. Importantly, cell type-specific CHD7-binding sites in hNCCs were enriched in SEs and predicted critical transcription factor-binding regions, such as TFAP2A, that drive hNCC-specific cell state-determining gene expression. Furthermore, the presence of CHD7 in hNCC transcription factor-binding regions increased the accuracy of detecting genes with hNCC-specific expression patterns, suggesting that profiling of CHD7-binding properties in hNCCs may provide an excellent resource for hNCC biology.

NCC populations arise transiently from the neural tube during development^41–43^. Importantly, they acquire broad differentiation and migration potentials and contribute to craniofacial bone, cartilage, peripheral nervous system, pigment cells, and specific heart structures^41–43^. Because NCCs contribute to the construction of the cranial mesenchyme, abnormal NC development is associated with various congenital malformations known as neurocristopathies^44^, which manifest as hearing loss and complex craniofacial defects and comprise one of the most common types of congenital disabilities^45^. Because the NC cannot be studied in the embryonic context, NCC induction systems using PSCs are essential for investigating the molecular mechanisms regulating the cellular behavior of NCCs, especially in humans^11,12^.

Based on this background, unbiased high-throughput transcriptomic and epigenomic profiling of hNCCs can be performed to identify cis- and trans-regulatory factors that control hNCC development^38,39,46,47^. The master lineage designator TFAP2A and the nuclear receptor NR2F1/2 simultaneously recognize the enhancer-coded sequence in hNCCs, leading to a transcriptionally permissive enhancer chromatin state in hNCCs^39^. The current analysis showed that these factors cooperated with CHD7 in hNCCs and that the regions co-occupied with TFAP2A, NR2F1/2, and CHD7 were highly linked to hNCC-specific gene expression (Fig. 5 and Supplementary Fig. S3). The presence or absence of CHD7 binding may thus predict the cell-specific genes essential for development and cell function in hNCCs.

We previously demonstrated the defective migration of iPSC-NCCs derived from CHARGE patients (CHARGE-iPSC-NCCs)^12^, and also reported 338 differentially expressed genes (DEGs) (238 upregulated and 100 downregulated) in CHARGE-iPSC-NCCs^12^. We therefore considered that it would be interesting to determine if the datasets obtained in the current study could help to elucidate the molecular mechanism underlying the cellular phenotypes of CHARGE-iPSC-NCCs. We performed analyses to integrate these data and found that, although CHD7-bound genes were expected to be downregulated in CHARGE syndrome, the downregulated DEGs were not included in the CHD7-bound gene lists, while about ten upregulated DEGs in CHARGE-iPSC-NCCs were included as CHD7-bound genes (data not shown).

Furthermore, we also examined the expression of genes bound by CHD7, TFAP2A, and NR2F1/2. Although a subset of genes was slightly downregulated in CHARGE-iPSC-NCCs, there was no major difference in the expression of these target genes (Supplementary Fig. S4). Further analyses should be performed to investigate this divergence in results in CHARGE-iPSC-NCCs.

One potential limitation of the current study was the axial-specific heterogeneity of the hNCCs. NC development progresses from anterior to posterior within the embryo during neurulation^41,42,48^, and despite their multipotency, NCCs produce different derivatives depending on their axial position^48^. Given that the hNCCs used in the current study were cranial NCCs^11,12^, and CHD7 may have different targets in other types of NCCs. Furthermore, this analysis compares multiple ChIP-seq data published by different groups using different antibodies. Therefore, it may have affected peak detection compared to the analysis that directly compares the signal intensity between each cell type.

Non-coding genomic mutations are implicated in human diseases, with associations between mutations in the enhancer region and the pathogenesis of disease progression^47,49,50^. Furthermore, mutations in tissue-specific enhancer regions can disrupt target-gene regulation in specific tissues. Indeed, mutations in the enhancer region of *SOX9*, an essential gene for NC development, have also been reported in the human craniofacial disorder Pierre Robin sequence^43^. Given that the impact of mutations in non-coding areas on long-term functional outcomes is difficult to address, the current results may help to determine the significance of genetic mutations in the enhancer regions in diseases considered to be caused by defects in NC development.

## Methods

### Cell culture

The hiPSC line 1210B2 was maintained as described previously^51,52^. The hiPSCs were dissociated using 0.5× TrypLE Select (Thermo Fisher) and plated on iMatrix511 (Nippi)-coated dishes in StemFit (AK03N) medium at feeder-free condition. The hiPSC-derived NECs were established and maintained as described previously^53,54^. The hNECs were passaged every 4 days and plated at a ratio of 1:5. The cells were dissociated using TrypLE Select (Thermo Fisher) and plated on Matrigel (Corning)-coated dishes in RHB-A medium (Takara) supplemented with 10 ng/mL EGF (Peprotech) and 10 ng/mL FGF2 (Peprotech). The hiPSC-derived NCCs were established previously and passaged less than three times for analysis. The hNCC medium consisted of 1:1 neurobasal medium (Thermo Fisher Scientific) and D-MEM/Ham’s F-12 (Wako) medium containing 1× GlutaMax (Thermo Fisher Scientific), 0.5× GEM 21 NeuroPlex serum-free supplement (Gemini Bio Products), 0.5× N2 supplement (Thermo Fisher Scientific), 5 mg/mL insulin (Sigma-Aldrich), 0.5% penicillin and streptomycin (Nacalai tesque), 20 ng/mL FGF2 and 20 ng/mL EGF. The hNCCs were dissociated using TrypLE Select and plated on fibronectin (Sigma-Aldrich)-coated dishes.

### Plasmid construction

Selected genomic regions near the human MSX2 and GPC1 genes were amplified by PCR from the human genomic DNA and cloned into a pGL3 promoter vector (Promega). Primers used for cloning were listed in Supplemental Table S4.

### Luciferase assay

hiPSCs, hNECs, and hNCCs were replated onto 12-well plates (Greiner Bio-One) and transfected with a reporter vector the next day. The sea pansy luciferase gene conjugated with human elongation factor-1α promoter (R-Luc) was also cotransfected as an internal control. Transfection was performed using a genejuice (Merck) according to the manufacturer’s procedures. Cells were solubilized 24 h after the transfection, and luciferase activity was measured according to the procedures recommended for the Dual-Luciferase Reporter Assay System (Promega) using GLOMAX multi-detection system (Promega). Firefly luciferase activity was divided by R-Luc activity from the cotransfected control plasmid.

### ChIP-reChIP qPCR

Chromatin immunoprecipitation was performed as previously described with minor modifications^10,39^. The dual cross-linking method was performed using 200 mM EGS for 30 min followed by 1% formaldehyde for 10 min and quenched by adding glycine to a final concentration of 125 mM. Cells were collected with NP-40 buffer (10 mM Tris–HCl, 10 mM NaCl, 0.5% NP-40) and centrifuged to a pellet, lysed in SDS lysis buffer (50 mM Tris–HCl, 1% SDS, 10 mM EDTA), and then incubated for 10 min on ice. Cell lysates were sonicated using Covaris S2 (Covaris) until the DNA fragments were 200—300 base pairs in length. These chromatin samples were diluted 1:10 with dilution buffer [50 mM Tris–HCl, 167 mM NaCl, 1.1% Triton X-100, 0.11%s sodium deoxycholate (NaDOc)], and 5% of the total volume was stored as input. The first immunoprecipitation was performed at 4 °C overnight with 3 μγ of an antibody conjugated with Dynabeads (VERITAS). Immune complexes were washed sequentially RIPA low-salt (50 mM Tris–HCl, 150 mM NaCl, 1 mM EDTA, 0.1% SDS, 1% Triton X-100, 0.1%NaDOc), RIPA high-salt (50 mM Tris–HCl, 500 mM NaCl, 1 mM EDTA, 0.1% SDS, 1% Triton X-100, 0.1%NaDOc), TE and eluted by TE/10 mM DTT at 37 °C for 30 min. Eluted chromatin was diluted 20 times with RIPA row-salt buffer for the second immunoprecipitation reaction. Next, immune complexes were washed, and immunoprecipitated DNA was eluted with elution buffer (10 mM Tris–HCl, 300 mM NaCl, 5 mM EDTA, 0.5% SDS) at 65 °C for 4 h. DNA was further incubated with RNase and proteinase K (Nacalai Tesque) and purified using a ChIP DNA clean and concentrator kit (Zymo Research). Antibodies used in this study have been reported as suitable for ChIP assay: anti-CHD7 (D3F5, Cell Signaling Technology), anti-TFAP2A (sc-12726, Santa Cruz Biotechnology), anti-NR2F1 (PP-H8132-00, Perseus Proteomics) and anti-NR2F2 (PP-H8132-00, Perseus Proteomics). Quantitative PCR (qPCR) analysis was performed using a ViiA7 system (Applied Biosystems) with KAPA SYBR FAST Master Mix (KAPA Biosystems). The primers used are listed in Supplemental Table S4.

### Reanalysis of ChIP-seq data

ChIP-seq data for histone modification in hNCCs were obtained from the Gene Expression Omnibus (GEO) database (GSE28876). Other ChIP-seq data for CHD7-binding in hNCCs and other ChIP-seq data for hiPSCs and hNECs were deposited as a part of our previous study through GEO accession number GSE108506. Raw reads were trimmed based on read length and quality using Trimmomatic (v. 0.33). The trimmed reads were then aligned to the reference genome (UCSC hg19) using Bowtie2 (v. 2.1.0) with the default parameters, and only uniquely mapped reads were used for subsequent analyses. The resulting SMA files were converted to the BAM format using SAMtools (v. 0.1.19). Peak calling was performed using MACS2 (v. 2.2.7.1) with default settings^55^. Cell type-specific CHD7-peaks were identified using the DiffBind (v. 3.6.1) R package. Overlaps for each peak were calculated using bedtools (v. 2.17.0) with default parameters. The genome-wide peak distribution from TSS was calculated using the ChIPseeker (v. 1.32.0) R package, and functional GO analysis of CHD7 peaks was performed using the GREAT website (http://bejerano.stanford.edu/great/public/html/index.php). The enhancer category was calculated based on the overlap of the CHD7-binding site and the enhancer region registered in the VISTA enhancer browser.

### Heatmap analysis

Heatmaps for each sample against the CHD7-binding region were made using deepTools (v. 1.5.11). The average H3K27ac read density at each region and the corresponding flanking region were calculated (bin size = 50). The lengths of the SE regions (between TSS and TES) were scaled relative to the median length.

### Motif search

Enriched sequences in the top 500 CHD7-binding regions were searched using MEME (v. 1.4) software and the identified sequences were compared using motif databases (JASPAR and HOCOMOCO) with TOMTOM (v. 2.2) software.

### Identification of SEs and associated genes

SEs were identified using the ROSE algorithm with default parameters based on H3K27ac intensity, in which enhancer peaks located within 12.5 kb were stitched together and ranked based on their input-subtracted signal for H3K27ac^31^. We used a promoter-exclusion zone of 5000 bp to prevent any enhancer contained within a window of ± 2500 bp around an annotated TSS from being stitched. Enhancer-associated genes were defined based on the calculated distance from the center of the SE to the nearest TSS of each gene. The assignment of enhancers to the closest genes in hNCCs is shown in Supplementary Table S2.

### Library preparation and RNA-seq

Samples for RNA-seq were prepared using the TruSeq RNA Library Prep Kit (Illumina) according to the manufacturer’s protocol. The sequencing library was sequenced on a NestSeq 1000 (Illumina). The adapter sequence and low-quality reads were removed using trimmomatic (v. 0.33), and the trimmed reads were mapped to reference genome hg38 using Kallisto (v. 0.46.2) with default settings. The count matrix data from Kallisto was loaded into R software (v. 4.2.0), and downstream analysis was performed. Differentially expressed genes were evaluated using the limma (v. 3.52.2) library.

### Computational analysis of CHARGE patient microarray data

Our previously published gene expression profiles of WT and CHARGE patient-derived NCCs data (GSE86212) were loaded into R (v. 4.2.0) for statistical analysis. Boxplots were made using the R package ggplot2 (v. 3.3.6).

## Supplementary Information

Supplementary Legends.

Supplementary Table S1.

Supplementary Table S2.

Supplementary Table S3.

Supplementary Table S4.

Supplementary Figures.

## Data Availability

RNA-seq data have been deposited in the NCBI GEO database (https://www.ncbi.nlm.nih.gov/geo/) and are accessible through the following GEO accession number (GSE220680).
