# Supplementary material for: Chromatin remodeler CHD7 targets active enhancer region to regulate cell type-specific gene expression in human neural crest cells
Source: Sci Rep. 2022 Dec 31;12:22648. doi: 10.1038/s41598-022-27293-6 (PMC9805427; doi:10.1038/s41598-022-27293-6)
Supplement: Supplementary file 1 — Supplementary Legends. [file 41598_2022_27293_MOESM1_ESM.docx]

**Supplemental Figure 1. CHD7 bound to cell type-specific regulatory regions**

(A) Distribution of CHD7-bound regions near gene body. The mean ChIP-seq signal was plotted. (B) CHD7-binding sites in hiPSCs, hNECs, and hNCCs. ChIP-seq peaks were detected using MACS2 peak caller (v. 2.2.7.1). Cell type-specific peaks were identified using DiffBind (3.6.1) R software. (C) Cell type-specific CHD7-binding sequences were analyzed and the top five motifs of each cell type were plotted.

**Supplemental Figure 2. Distribution of transcription factor binding sites in hNCCs**

(A) CHD7-, TFAP2A-, NR2F1-, and NR2F2-binding loci were distributed from TSS regions in hNCCs. (B) Heatmap analyses of the ChIP-seq signal of EP300 and selected histone modifications (H3K4me1, H3K4me3, H3K27ac, and H3K27me3). All ChIP-seq signals displayed from ±5 kb surrounding the center of each annotated CHD7, TFAP2A, NR2F1, and NR2F2 peak.

**Supplemental Figure 3. Gene expression of cell type-specific genes**

(A) Bar chart showing *CHD7, SOX2, PAX6, TFAP2A,* and *MSX2* gene expression in hiPSCs, hNECs, and hNCCs. (B) Heatmap of DEGs in each cell type. Expression levels were converted to z-score and plotted. (C) A number of detected DEGs and ChIP-seq target genes.

**Supplemental Figure 4. Gene expression of hNCC-specific genes in CHARGE-iPSC-NCCs**

Boxplot showing CHD7/TFAP2A/NR2F1/2-target gene expression (*CADM1*, *CBX7*, *PDLIM4*, *PLXND1*, *SNAI1*) in healthy control and CHARGE-iPSC-NCCs.

**Supplementary Table S1**

List of data sets of ChIP-seq in hiPSCs, hNECs and hNCCs analyzed in this study.

**Supplementary Table S2**

List of super-enhancer target genes in hNCCs. The genes closest to the super-enhancers center were listed as the target gene. The nearest CHD7-bound loci and VISTA enhancer-validated loci were listed.

**Supplementary Table S3**

List of cell type-specific target genes of CHD7 in hNCCs.

**Supplementary Table S4**

List of primers used in luciferase reporter vector construction and qPCR.
