# Supplementary material for: Chromatin remodeler CHD7 targets active enhancer region to regulate cell type-specific gene expression in human neural crest cells
Source: Sci Rep. 2022 Dec 31;12:22648. doi: 10.1038/s41598-022-27293-6 (PMC9805427; doi:10.1038/s41598-022-27293-6)
Supplement: Supplementary file 6 — Supplementary Figures. [file 41598_2022_27293_MOESM6_ESM.pdf]

Sanosaka et al., Supplementary Figure 1

A

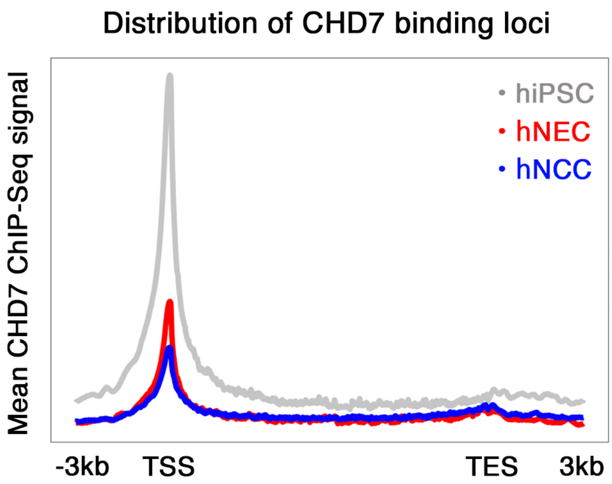

B

sample	# of peaks	# of cell type specific peaks
hiPSCs	31,518	25,676
hNECs	17,941	11,529
hNCCs	5,357	3,953

C

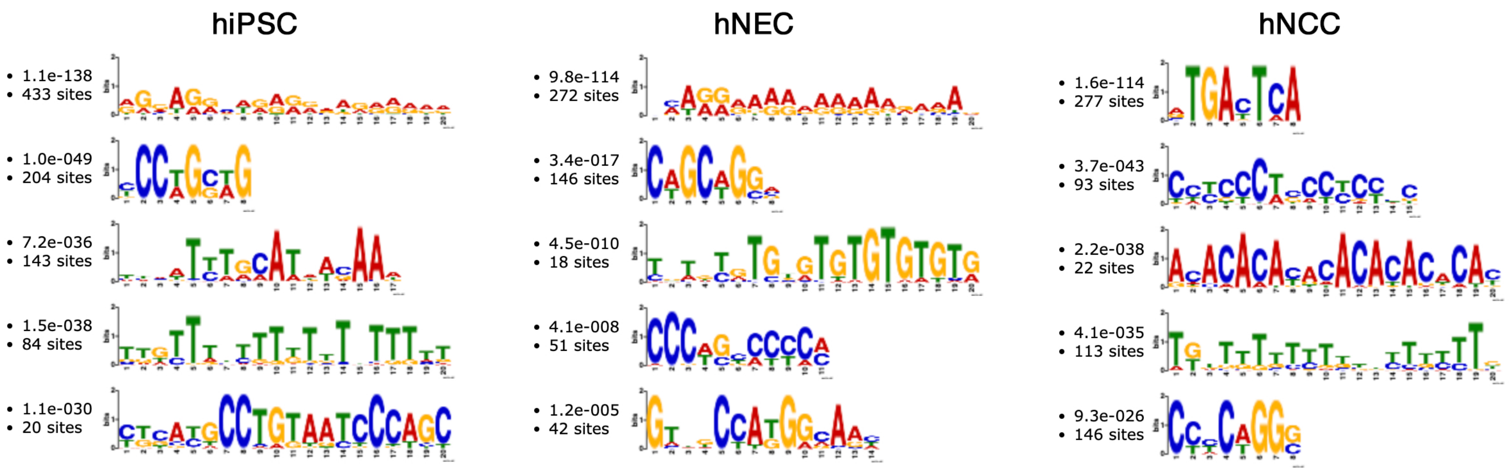

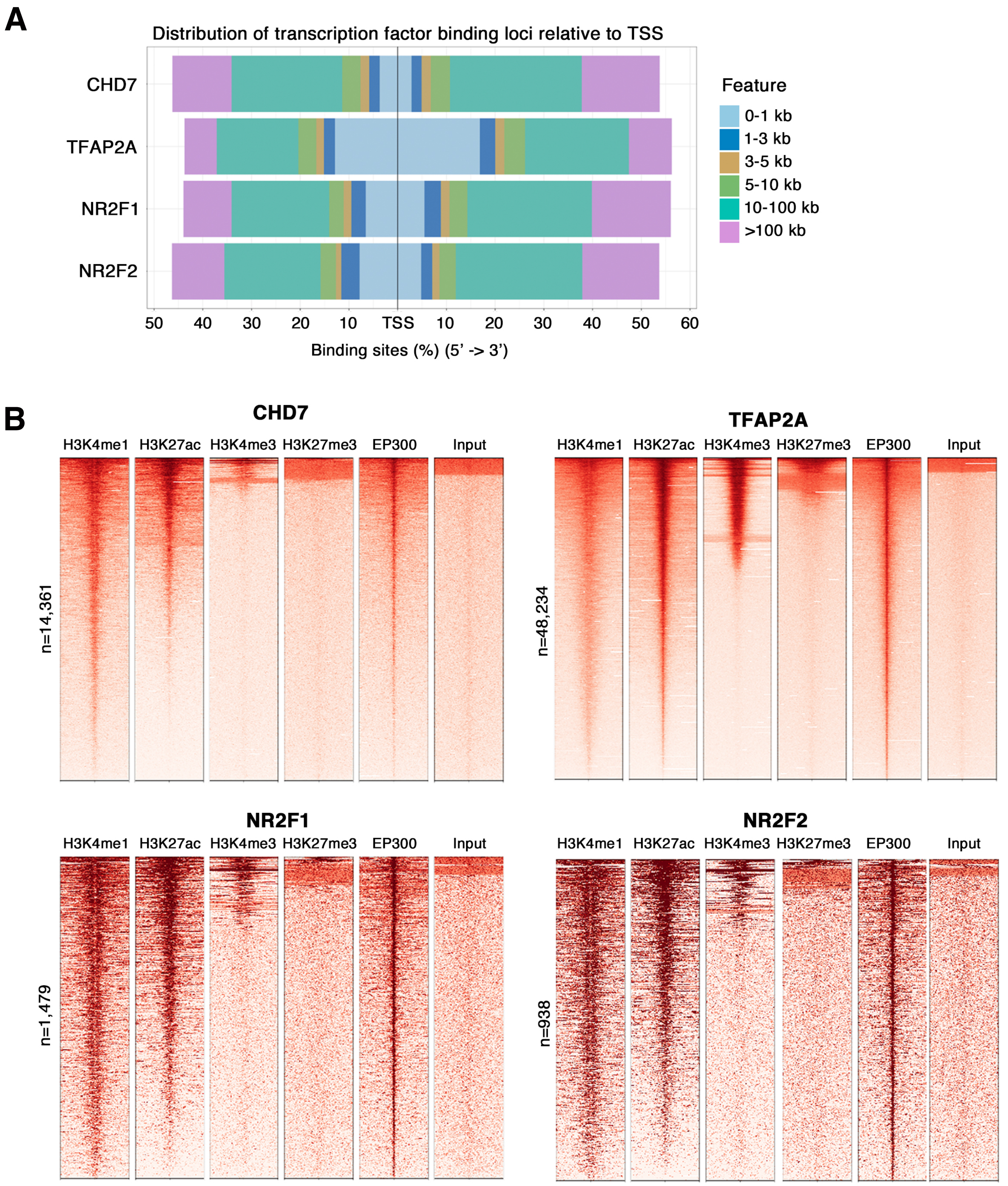
A
Distribution of transcription factor binding loci relative to TSS
CHD7
TFAP2A
NR2F1
NR2F2
50 40 30 20 10 TSS 10 20 30 40 50 60
Binding sites (%) (5' -> 3')
Feature
0-1 kb
1-3 kb
3-5 kb
5-10 kb
10-100 kb
>100 kb
B
CHD7
H3K4me1 H3K27ac H3K4me3 H3K27me3 EP300 Input
n=14,361
TFAP2A
H3K4me1 H3K27ac H3K4me3 H3K27me3 EP300 Input
n=48,234
NR2F1
H3K4me1 H3K27ac H3K4me3 H3K27me3 EP300 Input
n=1,479
NR2F2
H3K4me1 H3K27ac H3K4me3 H3K27me3 EP300 Input
n=938

A

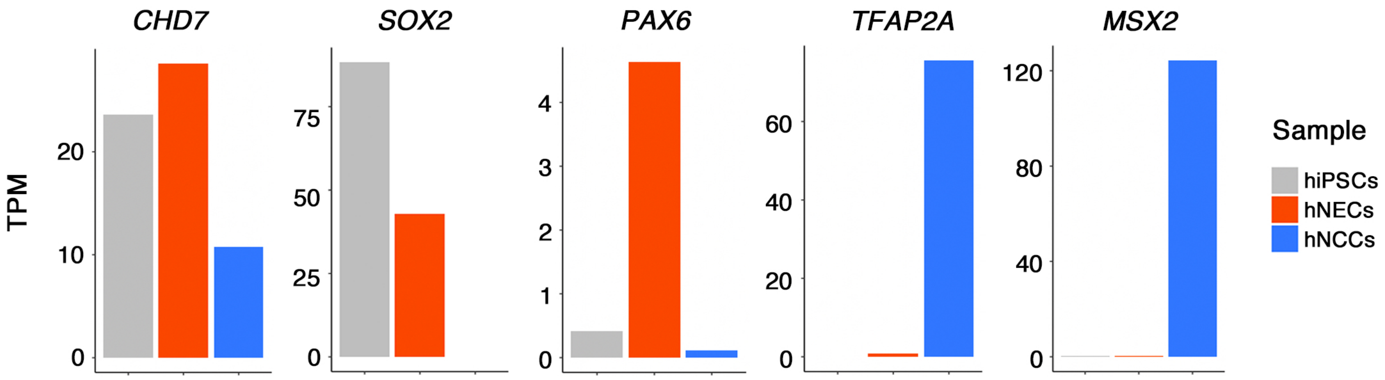

B

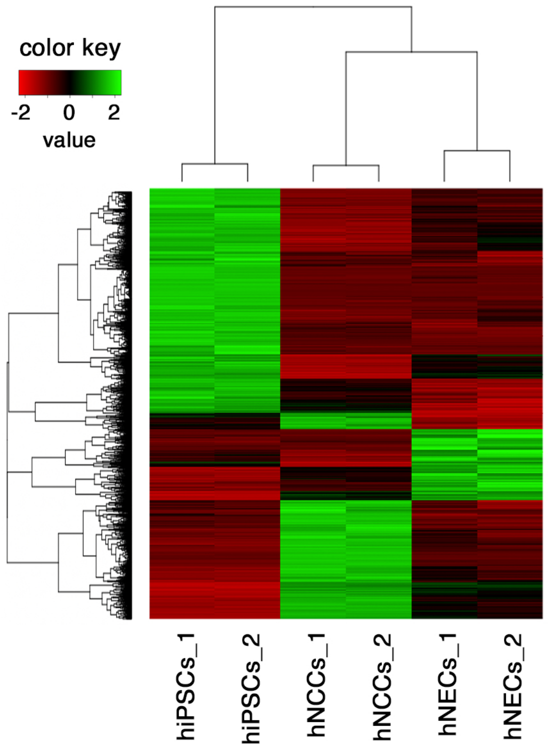

C

sample	ChIP-Seq target genes	ChIP-Seq (DEGs)
hiPSCs	2,766	3,228
hNECs	2,638	1,028
hNCCs	2,554	1,947

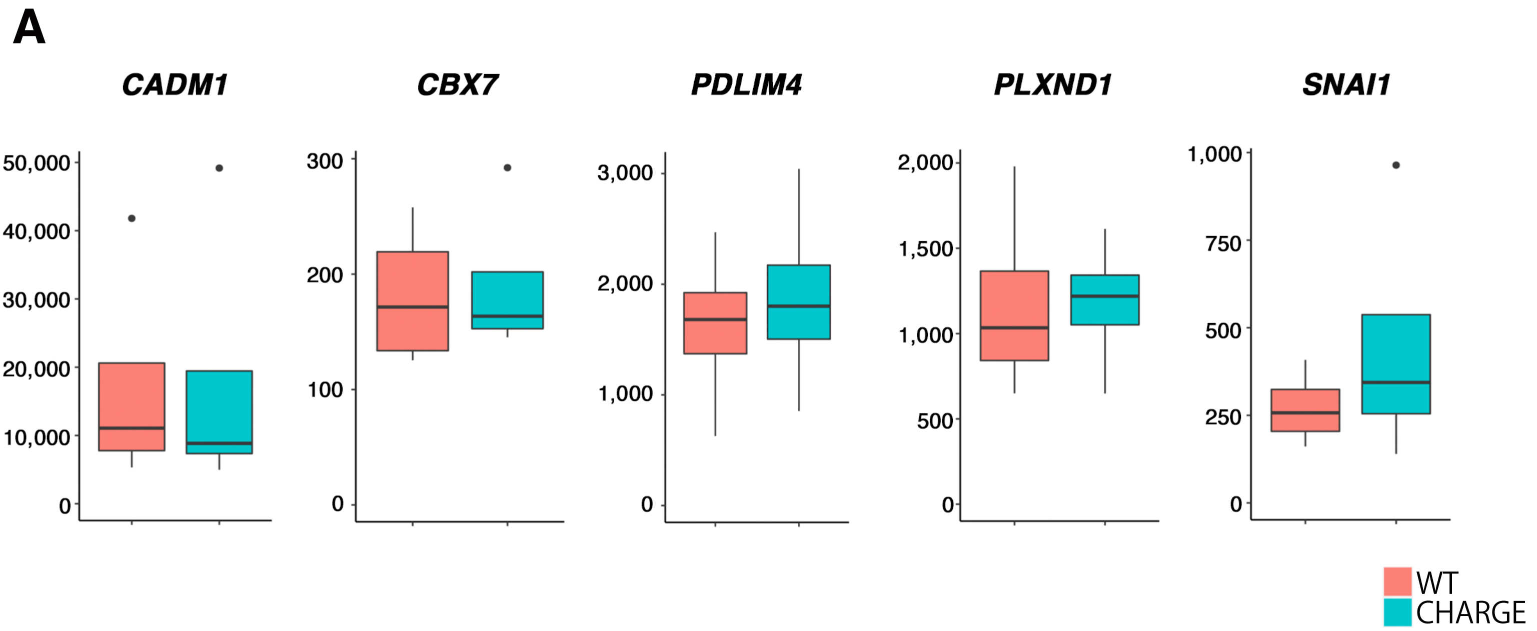
A
CADM1
CBX7
PDLIM4
PLXND1
SNAI1
50,000
40,000
30,000
20,000
10,000
0
300
200
100
0
3,000
2,000
1,000
0
2,000
1,500
1,000
500
0
1,000
750
500
250
0
WT
CHARGE
